# High levels of anti-Nef antibodies may prevent AIDS disease progression in vertically HIV-1-infected infants

**DOI:** 10.7448/IAS.17.1.18790

**Published:** 2014-02-14

**Authors:** Guillermo Corró, Cintia Milena Crudeli, Carlos Alberto Rocco, Silvia Alejandra Marino, Luisa Sen

**Affiliations:** 1 Laboratorio de Biología Celular y Retrovirus, Hospital de Pediatría “Prof. Dr. Juan P. Garrahan”, Buenos Aires, Argentina; 2 Consejo Nacional de Investigaciones Científicas y Tecnológicas (CONICET), Buenos Aires, Argentina

**Keywords:** antibodies, Nef, LTNP, paediatric AIDS, Nef-dependent cytotoxicity, apoptosis

## Abstract

**Introduction:**

HIV-1-associated CD4+ T-cell depletion is a consequence of uninfected cell death. Nef is one of the viral factors that trigger apoptosis on bystander cells, though the plasma Nef levels do not correlate with Th lymphocytes counts. The aim of our study was to evaluate whether anti-Nef antibodies were involved in paediatric AIDS development and whether they can prevent the CD4+ T-cell depletion in vertically infected children.

**Methods:**

Two hundred and seventy three HIV-1 vertically infected children seen at Garrahan Paediatric Hospital were randomly included in the study, adding 13 selected cases: seven LTNP (long-term non-progressors) and six RP (rapid progressors) children (*n*_total_=286). Specific anti-HIV-1-Nef antibodies were titrated by indirect ELISA and compared between groups. The plasma blocking effect on Nef-dependent cytotoxicity was evaluated in Jurkat cells using recombinant Nef as apoptotic stimulus and patient plasmas as blockers, measuring the apoptotic levels using Annexin-V stain and flow cytometry.

**Results:**

Only 63.4% of the patients had specific anti-Nef antibodies, and the levels of anti-Nef antibodies found in the selected LTNPs plasmas were always significantly higher (*p*=1.55×10^−4^) than those in RPs or general HIV-1+ paediatric populations. The LTNPs’ plasma had a strong inhibitory effect on Nef-dependent cytotoxicity even at high dilutions, while RP plasmas had little or no effect on Nef-induced apoptosis.

**Discussion and conclusions:**

High anti-Nef antibody levels are associated and predict slow or non-progression to AIDS in vertically HIV-1-infected children. They could be an efficient tool in preventing Nef-associated bystander effect, preserving CD4+ T-cells and the immune function in the context of paediatric HIV-1 infection.

## Introduction

Immunodeficiency associated with HIV-1 infection has been extensively studied and documented. The main cause of failure of the immune system is CD4+ T-cell depletion mostly as a result of uninfected T-cell death, known as the “bystander effect” [1, 2]. Although the exact mechanism underlying the bystander effect is still unclear, it is known that some viral proteins, such as gp120, Tat and Nef, may be responsible for the induction of uninfected cell death. Nevertheless, Nef is the only protein that has been associated with AIDS progression, as patients infected with ΔNef viruses either do not progress or progress slowly to AIDS [3–6].

Nef is a 27–32 kD myristoylated protein, encoded at the 3′ end of the HIV-1 genome. Many of its functions within the infected cell have been described. Among these, CD4 and MHC-I downmodulation has been associated with HIV-1 virulence [7, 8], both in adults [9, 10] and children [11, 12]. By sequestering CD4 from the cell surface, Nef prevents superinfection, allows virion budding and prevents immune system activation and cell-to-cell collaboration, while MHC-I downmodulation has been shown to be an effective mechanism to evade cytotoxic T-lymphocyte surveillance [13]. Nevertheless, none of these functions are directly associated with T-cell depletion.

In 1996, it was reported that Nef had a soluble form that could be detected in plasma from infected patients [14] and that it was able to exert a cytotoxic effect on bystander CD4+ T-cells *in vitro* [14–17]. There are few studies reporting measurements of Nef in plasma of HIV-1-infected individuals [14, 17], which suggests that Nef plasma levels do not correlate with CD4+ T-cell counts, as would be expected based on Nef-induced Th lymphocyte apoptosis. However, these studies also showed that anti-Nef antibodies inhibit Nef-induced T-cell death, indicating that anti-Nef antibodies *in vivo* could act as Nef blockers, preventing the bystander effect.

The aim of our study was to evaluate whether anti-Nef antibodies can prevent CD4+ T-cell depletion in vertically HIV-1-infected children. We observed that long-term non-progressor (LTNP) children always had high levels of antibodies against Nef and that those antibodies were able to block Nef-induced T-cell death *in vitro*.

## Methods

### Patients

All vertically HIV-1-infected children seen at “Garrahan” Paediatric Hospital in Buenos Aires, Argentina, between June 2006 and January 2007 were included in this study (here called non-LTNP, *n*=273). We included seven vertically infected LTNPs, older than 10 years, who had not received any antiretroviral therapy (ARVT) for at least eight years and who had always had CD4+ T-cell counts higher than 500 cells/mm^3^. Six rapid progressors (RPs), who developed AIDS before two years of age were also included (*n*_total_=286). These LTNPs and RPs were previously published by our group [11]. Epidemiological, immunological, and virological parameters of LTNPs and RPs are shown in Table 1.

**Table 1:** Features of our cohort of vertically HIV-1-infected children

Patient	Gender	Status	Age at sampling^a^ (months)	Age at ARV^b^ treatment beginning (months)	CD4+T-cell counts (cells/µl)^c^	Plasma viral load (Log_10_ RNA copies/ml)^c^
#513	M	LTNP	157	204^d^	754±116	2.88±1.01
#3467	M	LTNP	127	180	896±72	4.25±0.66
#4273^e^	M	LTNP	130	130^d^	903–	3.27–
#3070	M	LTNP	124	127	623±90	4.05±0.53
#3468	M	LTNP	92	105	1097±198	3.14±2.17
#2074	M	LTNP	124	124	604±53	2.5±0.91
#509	F	LTNP	95	143^d^	968±216	3.76±1.14
#193^f^	F	RP	6	8	215–	>5.9
#38^f^	M	RP	6	12	144–	>5.9
#12	M	RP	52	10	322±103	3.36±1.21
#3	M	RP	82	9	247±97	4.10±0.95
#92	F	RP	38	6	279±145	4.34±1.62
#104	M	RP	97	4	210±102	3.72±1.04
non-LTNP^g^	–	–	123±51	–	927±512	2.56±2.05

a For LTNPs, age at first sample available and tested is shown. Note that the first sample of all LTNPs was obtained before ARV therapy initiation

b ARV: anti-retroviral

c mean±SD of tested samples from each patient. The clinical and laboratory follow-up of all but one (#4273) of these patients was every six months after diagnosis. Plasma viral loads were determined using Branch DNA (ORGANON), COBAS Amplicor (ROCHE) or COBAS Taqman (ROCHE) methods. CD4+ T-cell counts were assessed by flow cytometry staining for CD3/CD4 and determining percentage of CD3/CD4+ cells

d LTNP that had never received ARV therapy

e only one sample was available for this LTNP

f patients #193 and #38 died at 8 and 12 months, respectively. Data on CD4+ T-cell counts and VL were available from only one sample of each. At the age of six months, the antibodies detected were more likely passive trans-placental rather than the patient's own antibodies

g mean±SD of all samples of the non-LTNPs studied. As shown, there were no significant differences in age or gender compared to LTNPs or RPs.

### Recombinant Nef proteins

Recombinant subtype B and subtype F Nef proteins were obtained as antigens for ELISA. Briefly, NL4-3 and a subtype F isolate *nef* alleles were directionally subcloned into a pET 22b+ vector (Novagen) to express the recombinant protein as inclusion bodies in *E coli* BL21 DE3. Inclusion bodies were dissolved in 8 M UREA and protein refolding was carried out by dialysis with Phosphate Buffer Solution (PBS) 0.5 mM 2-ME. Purification was performed with ion exchange with DEAE-sepharose in 50 mM Tris-HCl pH 8 using a 0–1 M NaCl gradient. Purity was tested by SDS-Page with silver staining. Circular dichroism spectroscopy was performed to evaluate the secondary protein structure, and the antigenicity was tested by ELISA using anti-HIV-1_JR-CSF_Nef Mabs (obtained from NIH AIDS Research and Reference Reagent Program).

### Indirect ELISA and titre calculation

Maxi-sorb 96-well plates (Nunc) were coated using 1.25 µg of 1:1 mix of subtypes B and F recombinant Nef proteins as most of the study population was infected with B/F recombinant forms having clade F *nef* genes [11]. Blocking was performed with PBS containing 1% dried milk. Plasma samples were diluted 1:50 and 1:200 and pre-incubated with blocking solution for 1 hour before use. Interaction was detected using an anti-human gammaglobulin Horseradish Peroxidase conjugate (DAKO). All samples and controls were tested in triplicate. The titre was measured in a single dilution (using the value obtained at a 1:200 dilution) with the following equation: titre=(Abs_corr_ sample plasma×12,000)/Abs_corr_ reference plasma, where Abs_corr_ sample plasma is the mean absorbance value of the sample, 12,000 is the reference plasma titre (calculated by end-point titration), and Abs_corr_ reference plasma is the absorbance value for the reference plasma in the same plate, all of them corrected by the blank. The assay cut-off value was calculated using 307 seronegative plasma samples under the conditions mentioned above (176–91 males and 84 females – samples from healthy adult blood donors and 131–59 males and 72 females – samples from healthy children seen at the hospital for causes not related to HIV-1 infection and with a mean of age of 102±47 months). Anti-Nef antibodies were detected just in one of the seronegative plasma samples. This positive plasma sample corresponded to an adult donor who was infected with HTLV-I. The cut-off absorbance was established at 0.120 using the mean+2 SD. All plasma samples with absorbance levels higher than 0.120 were considered positive and subsequently the titres were calculated.

### Inhibition of Nef-induced cytotoxicity by patients’ plasma

The inhibitory power of patients’ plasma on Nef-induced apoptosis was evaluated on Jurkat cells (ATCC TIB-152). Cells were maintained in 10% FBS, 2 mM L-Glutamine, RPMI 1640/HEPES (Gibco) with streptomycin/penicillin. Apoptosis induction protocols were modifications from those reported by James *et al*. [15]. Briefly, 10^6^ cells in 1.5 ml medium were added with patients’ plasma at 1/50 or 1/500 dilutions. Immediately after, the recombinant Nef protein was added at a final concentration of 0.1 µg/ml as an apoptotic stimulus. After a 24-hour incubation at 37°C, cells were harvested and stained with Annexin-V-FITC and Propidium Iodide (BD Pharmigen) following the manufacturer's instructions and then analyzed by flow cytometry (ARIA II BD). Cells were first gated on a SSC vs. FSC dot plot to exclude debris. The homogeneous cell population (Jurkat cell line) was analyzed for Annexin-V stain. Each condition was evaluated in triplicate, analyzing 10^4^ events per sample. Every FITC-positive event was considered as an apoptotic cell and the percentage of apoptotic cells in every condition analyzed was calculated. Plasma from LTNPs, RPs (only those who had anti-Nef antibodies) and 10 non-LTNPs(Ab+) was tested. Control of apoptosis was assessed using H_2_O_2_ and a non-apoptotic control was performed using Jurkat cells without any stimuli. A control with Nef without either patient or control plasma was prepared to determine the level of apoptosis exerted by Nef. A control of cells with plasma but without Nef was established in order to rule out any effect of plasma upon the cells. A final control was performed with 25 non-HIV-1 donor plasma samples and Nef.

### Statistical analysis

Differences in anti-Nef antibody levels between LTNPs and non-LTNPs or LTNPs and non-LTNPs(Ab+) as well as differences in the inhibition power of Nef-induced apoptosis between plasma from LTNPs and RPs were evaluated with the Mann-Whitney–Wilcoxon test. All tests were two-tailed with a significance level of 0.05.

The positive/negative predictive value and confidence intervals were estimated taking into account a case-control study design, according to the standard logit method proposed by Mercaldo *et al*. [18] and as implemented in the R Cran package “bdpv.”

## Results

### Comparison between anti-Nef antibody titres in LTNPs and in the general population of HIV-1-infected children

In order to evaluate whether the level of anti-Nef antibodies is a predictive factor for slow progression to paediatric AIDS, we developed an ELISA to detect and titrate the specific antibodies against Nef. Plasma samples of 286 vertically HIV-1-infected children were tested. We observed that only 63.8% (185/286) of the children had anti-Nef antibodies. All LTNPs had anti-Nef antibodies, while two out of the six RPs were negative for the anti-Nef antibodies. The absence of anti-Nef antibodies in 36.2% of the children tested was not associated with a lack of humoral immune response to HIV-1 or with agammaglobulinaemia since all patients had a positive Western blot for HIV (Bio-Rad) and more than one HIV-1 protein was recognized (data not shown). In addition, antibodies towards p24 were also evaluated in 72 patients of this cohort, including the seven LTNPs and the six RPs children as described by Allain *et al*. [19]. This demonstrated that only 59.5% had p24 serum binding capacity (43/72, 5/7 LTNP), which is in agreement with previous reports in this field [19–21] since most of the children included in this study had moderate to high viral loads, as shown in Table 1. Moreover, the anti-p24 titres obtained for LTNPs and non-LTNPs were similar, showing no statistically difference (data not shown).

**Figure 1:**
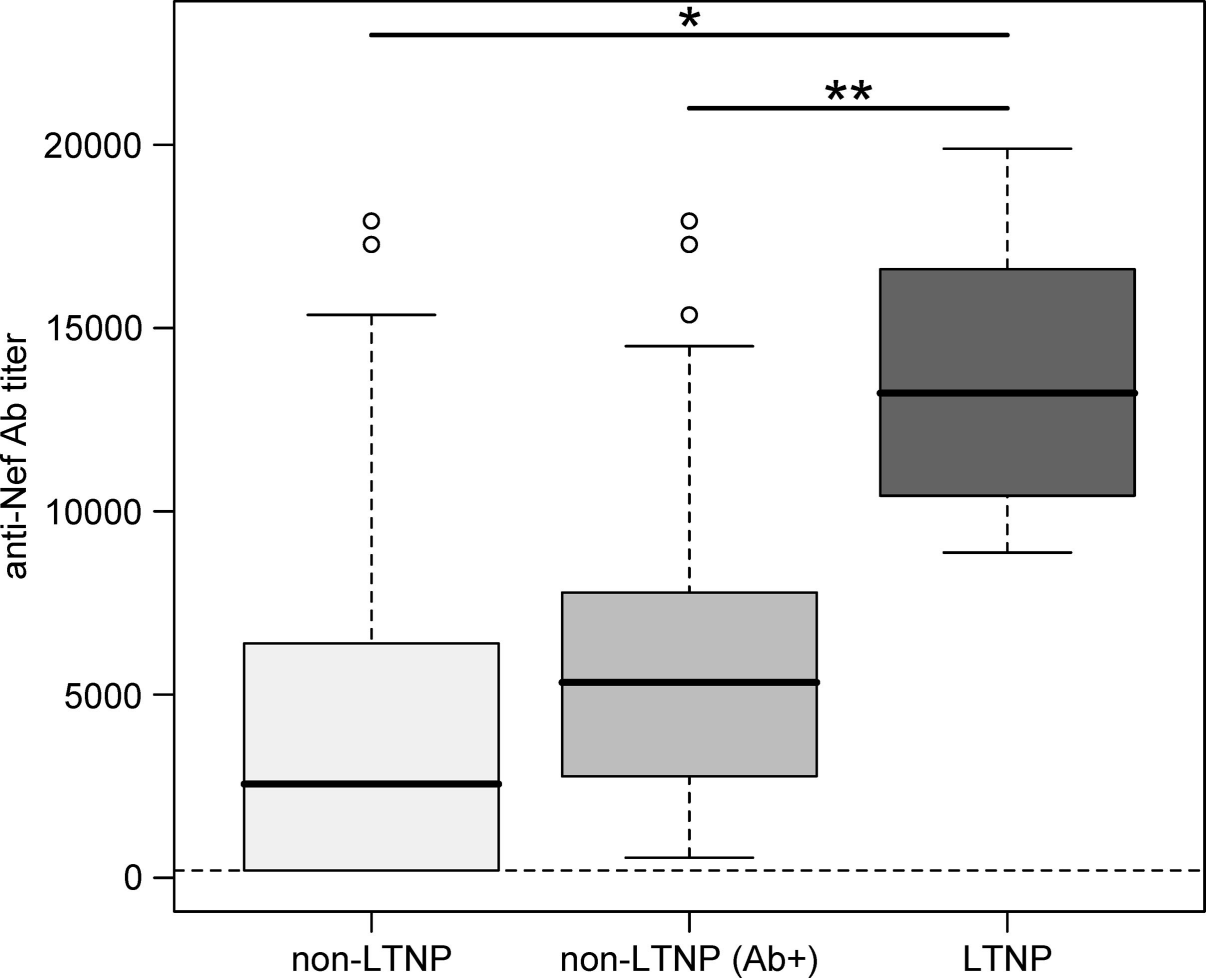
Comparison of anti-Nef antibody titres between different groups of HIV-1-infected children. Significant differences were observed between LTNPs (long-term non-progressors) and non-LTNPs (*p=2.955×10^−5^) and LTNP and those non-LTNPs who had antibodies against Nef [non-LTNP(Ab + )] (**p=1.55×10^−4^). The graph shows the analysis based on the titre mean of samples from each LTNP. Boxes represent the median and IQR for each group and subgroup of patients and data from percentile 5–95 are shown as points in the graph. *n*_LTNP_=7, *n*_non-LTNP_=279, *n*_non-LTNP(Ab + )_=177.

We classified the 286 patients into two groups according to the characteristics of their infection: LTNPs (as described in methods, *n*=7) and non-LTNPs (which included those who attended to the Hospital between June 2006 and January 2007 plus RPs, *n*=279). Subsequently, since 36.2% of non-LTNPs had no anti-Nef antibodies, we analyzed a subgroup of non-LTNPs who had a humoral immune response against Nef (*n*=177, 177/279) referred to as non-LTNPs(Ab+). Since several samples were available from each LTNP, the analyses were performed on the mean of all LTNP sample titres (*n*_samples_=34) or on the titre from the first sample available for each LTNP. As shown in Figure 1, the median difference of anti-Nef antibody titres was statistically significant between LTNPs and non-LTNPs and between LTNPs and non-LTNPs(Ab+) in both analyses (*p*_LTNPmean/Non-LTNP_=2.955×10^−5^/*p*_LTNPmean/Non-LTNP(Ab+)_=1.55×10^−4^ and *p*_LTNP first sample/Non-LTNP_=4.379×10^−5^/*p*_LTNP first sample/Non-LTNP(Ab+)_=2.691×10^−4^). The median titre for LTNPs was 13,722 (IQR: 10,053–18,346) when analyzing the titre mean of each LTNP and 15,116 (IQR: 11,733–19,200) when the first available sample of each LTNP was studied, while the median titre and IQR for non-LTNPs and non-LTNPs(Ab+) were 2454 (IQR: 50–6400) and 5333 (IQR: 2773–7893), respectively (Table 2). All LTNPs maintained high anti-Nef antibody titres at each time-point analyzed (between three and eight samples, Supplementary file 1), suggesting that the high level of antibodies against Nef is associated with a slow progression to paediatric AIDS.

**Table 2:** Anti-Nef antibody titres for all groups and subgroups analyzed

Patients	LTNP (mean)^a^	LTNP (first sample)^b^	Non-LTNP	Non-LTNP(Ab+)
Ab titre mean	13,722^c^	15,116^c^	2454	5333
IQR	10,053–18,346	11,733–19,200	50–6400	2773–7893

a Median titre calculated using all of the LTNP sample titres measured (between three and eight samples for each LTNP, *n*_samples_=34)

b median titre median calculated using the titre of the first chronological sample available for each LTNP

c both median titres differ significantly from median titres of non-LTNP and non-LTNP(Ab + ) as shown in Figure 1.

Setting the titre cut-off at 11,733, the lower quartile for LTNPs in the first sample would allow for the prediction of 6/7 LTNPs with a 94.6% specificity. Given that in our paediatric cohort an incidence of LTNPs of 2.3% was observed [11], the positive predictive value for a titre higher than 11,733, based on specificity and sensitivity, was 27.3% (95% CI: 17.2%–40.4%). Likewise, the negative predictive value would be 99.6% (95% CI: 97.9%–99.9%). The first available sample titre for LTNPs was chosen for this analysis because the prediction of long-term non-progression or rapid progression in children should be made as early as possible.

Three non-LTNP(Ab+) samples had similar high anti-Nef antibody titres to those observed for LTNPs. Only two of them had been followed at the Garrahan Hospital and their clinical charts were available. Both of them were vertically infected, were older than 12 years and had been free of HIV-1-related symptoms with normal CD4 T-cell counts according to age (Supplementary file 2). However, both had received ARVT at early ages and thus could not be considered LTNPs. The remaining patient was lost to follow-up and his history of disease could not be analyzed.

### *In vitro* inhibition of Nef-dependent cytotoxicity by plasma from LTNPs and RPs

**Figure 2:**
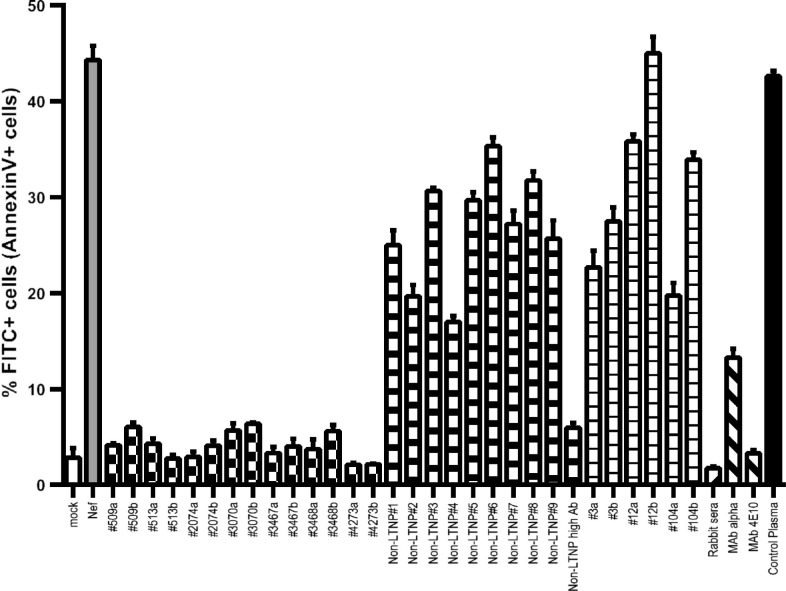
Plasma Inhibitory power on Nef-induced apoptosis. Mean and SD of apoptosis percentages (Annexin-V + cells/100 total cells) for Jurkat cells stimulated with recombinant Nef (grey bar) and protected with plasma of long-term non-progressors (LTNP, checkered bars), non-LTNP samples at the 1:50 dilution (non-LTNP #1–9 and non-LTNP high Ab, wide striped bars) rapid progressor (RP) plasma (thin striped bars), anti-Nef rabbit sera (to the right, diagonally striped bar), anti-Nef monoclonal antibody producing hybridoma supernatant (to the left, diagonally striped bars) or a mean of control HIV-1-negative plasma (black bar). Each bar is labelled below as #patient code “a” (plasma dilution 1:50) or “b” (plasma dilution 1:500).

The inhibition of Nef-induced apoptosis was evaluated *in vitro* as described in Methods. We compared apoptosis levels in each condition. The percentage of Annexin-V-positive cells that arose in the Jurkat culture treated with Nef (Figure 2, grey bar) was 44±2%. Cell cultures with LTNP plasma at the two dilutions tested (1:50 and 1:500) showed strong protection against cytotoxicity, with levels of apoptosis between 2 and 7% (Figure 2, checkered bars). Although plasma from RPs had anti-Nef antibodies, they showed poor or no effect on Nef-dependent cytotoxicity (Figure 2, thin striped bars) as did nine randomly selected plasma samples from non-LTNP(Ab+)s (Figure 2, wide striped bars). The plasma of one non-LTNP(Ab+) that had a high titre of anti-Nef antibodies, similar to that of LTNPs described in the preceding section, was also tested and showed strong protection against cytotoxicity, as was seen in LTNPs. Non-human anti-Nef antibodies were also able to block the cytotoxic effect of Nef *in vitro* (Figure 2, ascendant and descendent diagonally lined bars). The addition of 25 HIV-1-negative human plasma samples (randomly selected from the plasma of 131 children used to calculate the ELISA cut-off value) had no effect on Nef-induced cytotoxicity (Figure 2, black bar shows the mean of 25 triplicate determinations). Although the samples analyzed were few, the blocking effect of LTNP plasma was statistically higher than that observed for RP plasma (*p*<0.01).

## Discussion

We found that plasma from LTNP children had higher titres of anti-Nef antibodies than typical and RP children, suggesting that the high level of specific antibodies against Nef predicts slow or no progression to paediatric AIDS. We also demonstrated that LTNP plasma had a strong inhibitory power on Nef-induced apoptosis that was absent in RP or typical progressor (non-LTNP) plasma. This finding may partially explain the relationship between anti-Nef antibodies and non-progression to AIDS. Thus, the inhibition of Nef-dependent cytotoxicity on CD4+ T-cells could be a protective factor preventing the bystander effect and, as a consequence, T-lymphocyte depletion as observed in LTNPs, even in those who have persistently detectable and high viral loads as shown in Table 1.

The levels of the soluble form of Nef in HIV-1-infected plasma range from 1 to 100 ng/ml [14, 17]. At these concentrations, Nef exerts an *in vitro* cytotoxic stimulus on CD4+ T-cells [14–16]. It is reasonable to assume that with chronically high levels of Nef, the continuous apoptotic stimulus on CD4+ T-cells would lead to low T-lymphocyte counts and finally to T-cell depletion and AIDS. There have been few reports on Nef concentration measurement in plasma of HIV-1-infected individuals [14, 17]; however, contrary to expectation, these reports agreed that plasma Nef levels do not correlate with CD4+ T-cell counts. Based on our results, we postulate that specific antibodies against Nef may act as blockers neutralizing its effect in the extracellular medium, preventing plasma Nef from exerting a detrimental effect upon CD4+ T-cells.

No correlation between antibody levels and viral load or CD4+ T-cell counts was observed. This may have two possible explanations. We performed a cross-sectional study, and thus only one sample was available for each patient (except for the LTNPs). Therefore, we were not able to determine previous antibody levels. Since intermittency of anti-Nef antibody titres has been reported [22, 23], the correlation should be assessed with a longitudinal study design. The second main point is that the correlation should be analyzed not with antibody levels or Nef concentration alone, but with the ratio between these two parameters as we are looking for a correlation in a scenario that has more than two variables, i.e. T-cells, Nef and anti-Nef antibodies.

To the best of our knowledge, our study is the first to report anti-Nef antibody levels of vertically HIV-1-infected children and LTNPs. The percentage of HIV-1-infected children who had a humoral immune response against Nef was similar to that reported in adults [22–25], but we observed no anti-Nef antibodies in healthy individuals. Other authors have documented that the lack or intermittency of anti-Nef antibody levels is associated with rapid AIDS progression [22, 23]. On the other hand, we observed that children from our cohort with high titres of antibodies against Nef have a 10-fold higher probability of remaining asymptomatic for more than 10 years. Thus, anti-Nef antibody titre determination may be an efficient tool for the early detection of LTNP children.

We showed that high anti-Nef antibody titres are significantly associated with slow or no progression to paediatric AIDS (*p*<0.001) and may neutralize the bystander effect associated with Nef, preventing T-cell death *in vitro*. Nevertheless, we cannot rule out that the quality and not the quantity of anti-Nef antibodies plays a role in preventing CD4+ T-cell death, since no dilution effect was seen in the cytotoxicity-inhibition assay for LTNP samples. So far, no reports directly assessing this issue have been published.

Our results also suggest that passive immunization against Nef may provide an efficient alternative therapy and that immunization against Nef for individuals at risk of infection may be a potentially good therapeutic vaccine.

## Conclusions

Anti-Nef antibody titre is associated with slow progression to paediatric AIDS, with interesting therapeutic perspectives. The role of anti-Nef antibodies in delaying progression towards AIDS may be related to the capacity to prevent Nef-induced apoptosis *in vitro*. Furthermore, statistical analysis showed that the measurement of anti-Nef antibody titre would be a potential predictive factor for non-progressive vertical HIV-1 infection.
